# Polymorphism in one-carbon metabolism pathway affects survival of gastric cancer patients: Large and comprehensive study

**DOI:** 10.18632/oncotarget.3259

**Published:** 2015-03-25

**Authors:** Tingting Zhao, Dongying Gu, Zhi Xu, Xinying Huo, Lili Shen, Chun Wang, Yongfei Tang, Peng Wu, Jason He, Weida Gong, Ming-Liang He, Jinfei Chen

**Affiliations:** ^1^ Department of Oncology, Nanjing First Hospital, Nanjing Medical University, Nanjing, China; ^2^ Department of Surgery, Yixing People's Hospital, Yixing, China; ^3^ College of Letter and Sciences, University of California at Berkeley, CA, USA; ^4^ Department of Surgery, Yixing Cancer Hospital, Yixing, China; ^5^ Stanley Ho Center for Emerging Infectious Diseases, and Li Ka Shing Institute of Health Sciences, Faculty of Medicine, The Chinese University of Hong Kong, Hong Kong, China; ^6^ Department of Biomedical Sciences, City University of Hong Kong, Hong Kong, China; ^7^ Collaborative Innovation Center for Cancer Personalized Medicine, Nanjing Medical University, Nanjing, China

**Keywords:** one-carbon metabolism (OCM), single nucleotide polymorphism (SNP), gastric cancer

## Abstract

Although it has been shown that polymorphisms in one-carbon metabolism (OCM) pathway are associated with gastric cancer (GC), their interactions and contributions for patients’ survival are elusive. In this study, we investigated the effects of polymorphisms and their interactions on the survival of GC patients, including genes of *Methylenetetrahydrofolate reductase* (*MTHFR 677C > T, 1298A > C*), *Methionine synthase reductase* (*MTRR 66A > G*), *Methionine synthase* (*MTR 2756A > G*), and *Thymidylate synthase* (*TS* 3′-UTR *ins6 > del6*, 5′-UTR *2R > 3R*). We recruited 919 GC patients from 1998 to 2006. The Kaplan–Meier plots, Cox regression analyses and the log-rank tests were carried out in this study. *MTHFR 1298CC* genotype showed protective effect (HR = 0.444, 95% CI = 0.210–0.940). *MTRR 66 GA + GG* genotypes decreased the risk of death (HR = 0.793, 95% CI = 0.651–0.967) in general, and in subgroups with more pronounced diffuse type, greater depth of invasion (T2/T3/T4), higher level lymph node metastasis (N1/N2/N3), advanced TNM stages (II/III level) and 5-Fu treatment. However, the improved survival disappeared when GC patients simultaneously had *MTR 2756 GA + GG* genotypes (HR = 1.063, 95% CI = 0.750–1.507). Although *MTRR 66GA* genotype was not associated with the survival of GC patients, patients with simultaneous *MTRR 66GA* and *MTR 2756AA* genotypes exhibited significant risk reduction of death (HR = 0.773, 95% CI = 0.609–0.981). *MTHFR 1298 CA + CC* combined with *TS* 5-UTR *2R3R + 3R3R* genotypes (HR = 0.536, 95% CI = 0.315–0.913) also increased patient survival rates. Our results suggest that the *MTRR 66A > G* and *MTHFR 1298A > C* polymorphisms may be useful prognostic biomarkers for GC patients.

## INTRODUCTION

Gastric cancer (GC), the fourth most common and the second most deadly cancer worldwide, is particularly prevalent in developing countries [1]. Although significant progress has been achieved in multimodal therapy strategies, such as combination therapy with surgery, chemotherapy, radiotherapy, or targeted therapy, the prognosis still remains poor with 5-year overall survival rates less than 25% [2]. Biomarkers for early diagnosis are important in deciding therapeutic options, as well as improving treatment efficiency and prognosis prediction [3].

Genetic factors play crucial roles in initiation of carcinogenesis and cancer development. Although gastric carcinogenesis undergoes highly complicated processes (e.g., abrogated gene expression, abnormal cell proliferation, resistance to apoptosis, dedifferentiation, enhanced survival, escape of immune surveillance, and metastasis) [4, 5], abrogation of gene expression or function caused by genetic changes (i.e., deletion, amplification and mutation) is the main force driving normal gastric cells into cancer cells. It has been shown that dysfunctions of one-carbon metabolism associated genes contribute to carcinogenesis via affecting DNA methylation, synthesis and repair [6]. One-carbon metabolism (OCM) pathway is a centered pathway involved in folate metabolism and DNA synthesis. It contains several crucial enzymatic reactions from folate uptake to synthesis of S-adenosylmethionine (SAM) [7]. Methylenetetrahydrofolate reductase (MTHFR) is a vitamin B2-dependent enzyme that carries out an irreversible conversion of 5,10-methylene-tetrahydrofolate (5,10-MTHF) to 5-methyl-tetrahydrofolate (5-MTHF) [8]. This is the rate-limiting step for OCM because 5,10-MTHF is the substrate for three other enzymatic reactions. Methionine synthase (MTR) catalyzes the remethylation of homocysteine to methionine [9], the residue critical for maintaining adequate intercellular folate level. Methionine synthase reductase (MTRR) maintains MTR in its active form. Thymidylate synthase (TS) catalyzes the reductive methylation of dUMP by 5,10-MTHF to form dTMP and dihydrofolate, a rate-limiting step in DNA synthesis [10]. TS is an essential enzyme in proliferating cells and is also an important target for a variety of chemotherapeutic drugs, e.g. 5-FU.

Previous studies have identified that functional polymorphisms of these genes in the OCM pathway were associated with human cancers, including colorectal cancer, head and neck cancer, esophagus cancer, hepatocellular cancer, lung cancer, renal cell carcinoma and gastric cancer [11–18]. Common variants of *MTHFR* (rs1801133, *677 C > T*, Ala222Val; and rs1801131, *1298 A > C*, Glu429Ala) reduced enzyme activity and plasma folate levels, resulting in hypomethylation [19]. Suboptimal methyl supply led to dysfunction of DNA methylation, a process highly associated with GC etiology [20]. The polymorphisms *of MTR* (rs1805087, *2756 A > G*, Asp868Gly) and *MTRR* (rs1801394, *66A > G*, ILe22Met), which were associated with hyperhomocysteinemia [21], impaired remethylation of homocysteine to methionine. The copy number of 28-bp tandem repeats in the 5′-untranslated region (5′-UTR) of *TS* gene affected the translational efficiency [22]. In a reporter assay, the 5′-UTR of *TS* with 3-repeats (3R) displayed much higher luciferase activities than that with 2-repeats (2R), suggesting this tandem repeats may enhance the assembly of translational machinery or mediate more effective binding between ribosome and *TS* mRNA [23]. It was shown that intratumoral TS protein level was associated with the sensitivity of chemotherapy (e.g. 5-FU) [24, 25]. A novel polymorphism with 6-bp deletion of the sequence TTAAAG at nt1494 was found to be associated with decreased *TS* mRNA stability in the 3-untranslated region (3′-UTR) of *TS* gene. Therefore, the deletion suppressed its expression [26, 27]. However, whether the 5′-UTR and 3′-UTR polymorphisms interact each other and display synergistic or counteractive effects on GC have not been investigated.

The effects of polymorphisms of individual gene or combinations of two genes have been investigated on GC survival. Whether these polymorphisms would interact with each other and display certain synergistic effects have not been reported. In this study, for the first time, based on large-number clinical data analysis, we systematically investigated the comprehensive effects of polymorphisms of four genes involved in OCM pathway on the clinical outcomes of GC patients.

## RESULTS

### Clinical features of the study subjects

The clinical characteristics of 919 retained GC patients were described in Table 1. The median age of the study subjects was 62.0 years (range, 28–83 years). All of the GC patients underwent the surgical resections and 297 patients received chemotherapy. 426 patients died during the 119.0 months of follow-up. The survival time was significantly related to varieties in tumor size, histological type and depth of invasion, lymph node metastasis and TNM stage (all log-rank *p* < 0.05). In particular, patients with tumor size >5 cm (MST, 50 months) had a 41% significantly higher risk of death (HR = 1.41, 95% CI = 1.16–1.71, *p* < 0.001) when compared with those with tumor size ≤ 5 cm (MST, 98 months). The intestinal-type patients (MST, 77 months) had a 64% significantly lower risk of death (HR = 1.36, 95% CI = 1.13–1.54, *p* < 0.001) than diffuse-type patients (MST, 62 months). Patients with lymph node metastasis (MST, 46 months) also had a 76% higher risk of death (HR = 1.76, 95% CI = 1.43–2.16, *p* < 0.001) when compared to those with no lymph node metastasis (MST, 80 months). Furthermore, as the depth of invasion and TNM stage increased, the risk of death also exhibited an obvious increase (log-rank *p* < 0.001).

**Table 1:** Gastric cancer patients’ characteristics and clinical features

Variable	Patients (*n* = 919)	Deaths (*n* = 426)	MST (months)	Log-rank *p*	HR (96% CI)^a^
Age (years)					
≤60	432	198	89	0.372	1.00
>60	487	228	62		1.090 (0.90–1.32)
Sex					
Male	706	323	74	0.384	1.00
Female	213	103	63		1.10 (0.88–1.38)
Tumor size					
<5 cm	570	241	98	< 0.001	1.00
>5 cm	349	185	50		1.41 (1.16–1.71)
Tumour site					
Non-cardia	313	141	77	0.354	1.00
Cardia	606	285	67		0.91 (0.74–1.11)
Histological type					
Intestinal	393	152	77^b^	< 0.001	1.00
Diffuse	526	274	62		1.36 (1.13–1.54)
Depth of invasion					
T1	177	56	86^b^	< 0.001	1.00
T2	134	59	78		1.52 (1.09–2.19)
T3	6	3	70		1.46 (1.23–2.37)
T4	583	295	52		1.91(1.43–2.54)
Lymph node metastasis					
N0	362	129	80	< 0.001	1.00
N1/N2/N3	546	290	46		1.76 (1.43–2.16)
Distant metastasis					
M0	903	417	70	0.437	1.00
M1	15	8	47		1.32 (0.66–2.65)
TNM stage					
I	239	80	84^b^		1.00
II	198	80	70	< 0.001	1.29 (0.92–1.77)
III	452	248	41		2.03 (1.57–2.61)
IV	22	12	40		2.13 (1.16–3.91)
Chemotherapy					
No	613	285	74	0.450	1.00
Yes	297	135	60		1.08 (0.88–1.33)

a Adjusted for age and sex.

b Mean survival time was provided when MST could not be calculated.

c Information was not available for two patients.

### Prolongation of patient survival with *MTRR 66A > G* polymorphism

**Figure 1:**
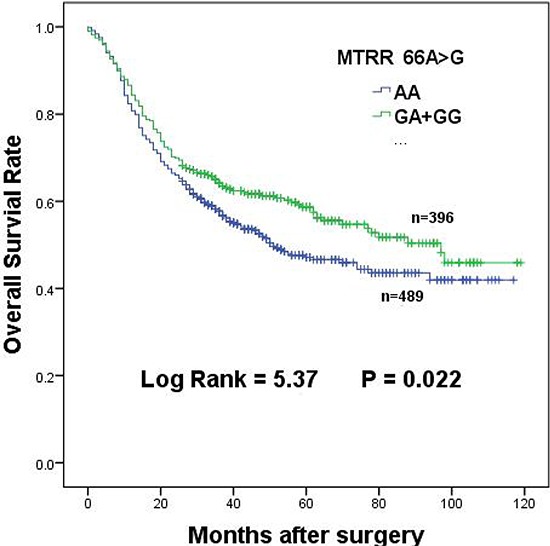
Overall survival of *MTRR 66A > G* dominant genotypes in gastric cancer patients

The relationships between individual polymorphisms and survival of GC patients in different genetic models were assessed by Cox regression analyses. Codominant, dominant and recessive models were applied in this study (Table 2). We first observed that *MTRR 66GG* genotype significantly protected GC patients from death. The MST of patients with GG genotype was extended from 51 to 87 months (HR = 0.500, 95% CI = 0.291–0.856, *p* = 0.014) when compared to those with *AA* genotype. A similar result was also obtained in a recessive model (HR = 0.537, 95% CI = 0.315–0.915, *p* = 0.019). Further analysis of *MTRR 66A > G* polymorphism in the dominant model revealed a remarkably longer survival time in GC patients (MST, 97.0 months; HR = 0.793, 95% CI = 0.651–0.967, *p* = 0.022) as compared to that in *AA* homozygote (Table 2). The overall survival of GC patients with *66A > G* dominant genotypes (*AG + GG*) was presented in Figure 1. Heterozygote *66A > G* genotypes exhibited marginal prolongation of survival of GC patients (HR = 0.883, 95% CI = 0.684–1.028, Table 2).

**Table 2:** Genotypes of *MTRR, MTHFR, MTR and TS* polymorphisms and gastric cancer patients’ survival

Genetic model	Genotypes	patients	Deaths	MST (months)	log-rank P	HR (95%CI)^a^
**MTRR rs1801394 A66G**						
Codominant model	AA	489	242	51	0.014	1.00
	GA	347	151	80		0.838(0.684–1.028)
	GG	49	14	87^b^		0.500(0.291–0.856)
Dominant model	AA	489	242	51	0.022	1.00
	GA/GG	396	165	97		0.793(0.651–0.967)
Recessive model	AA/GA	836	393	65	0.019	1.00
	GG	49	14	87^b^		0.537(0.315–0.915)
**MTHFR rs1801131 A1298C**						
Codominant model	AA	640	301	65	0.090	1.00
	CA	236	110	63		0.985(0.792–1.225)
	CC	28	7	81^b^		0.444(0.210–0.940)
Dominant model	AA	640	301	65	0.429	1.00
	CA/CC	264	117	89		0.918(0.742–1.137)
Recessive model	AA/CA	876	411	65	0.042	1.00
	CC	28	7	81^b^		0.446(0.211–0.942)
**MTHFR rs1801133 C677T**						
Codominant model	CC	313	151	60	0.793	1.00
	TC	438	200	78		0.940(0.761–1.161)
	TT	141	63	63		0.918(0.684–1.231)
Dominant model	CC	313	151	60	0.51	1.00
	TC/TT	579	263	74		0.935(0.766–1.143)
Recessive model	CC/TC	751	351	65	0.708	1.00
	TT	141	63	63		0.951(0.727–1.244)
**MTR rs1805087 A2756G**						
Codominant model	AA	684	314	52	0.041	1.00
	GA	149	69	88		1.033(0.796–1.341)
	GG	7	0	75^b^		0
Dominant model	AA	688	317	67	0.038	1.00
	GA/GG	149	69	70		0.972(0.749–1.261)
Recessive model	AA/GA	830	386	65	0.053	1.00
	GG	7	0	75^b^		0.049(0.001–3.620)
**TYMS 5-UTR 2R > 3R**						
Codominant model	2R2R	43	24	35	0.279	1.00
	2R3R	276	113	70		0.807(0.522–1.248)
	3R3R	51	27	78		0.732(0.481–1.115)
Dominant model	2R/2R	43	24	35	0.184	1.00
	2R3R/3R3R	794	357	74		0.758(0.501–1.146)
Recessive model	2R2R/2R3R	319	154	62	0.216	1.00
	3R3R	518	227	78		0.880(0.717–1.080)
**TYMS 3-UTR 6bp ins/del**						
Codominant model	I/I	61	30	46	0.682	1.00
	D/I	410	193	63		0.948(0.645–1.393)
	D/D	413	187	77		0.880(0.598–1.294)
Dominant model	I/I	61	30	46	0.63	1.00
	DI/DD	823	380	70		0.913(0.630–1.324)
Recessive model	II/DI	471	223	63	0.407	1.00
	D/D	413	187	77		0.922(0.759–1.119)

a Adjusted for age and sex.

b Mean survival time was provided when MST could not be calculated.

### *MTHFR 1298CC* genotype associate with decreased risk of death

In the case of *MTHFR 1298A > C* polymorphism, patients with homozygote *CC* genotype (MST, 81 months) had a 44% significantly lower risk of death (HR = 0.440, 95% CI = 0.210–0.940) when compared with those with *AA* genotype (MST, 65 months). A similar result was also obtained in a recessive model (HR = 0.446, 95% CI = 0.211–0.942, Table 2).

In the case of *MTHFR 677C > T* and other polymorphisms (*MTR 2756A > G*, *TS* 5′-UTR *2R > 3R* and 3′-UTR 6bp *ins > del*), no significant associations were identified between survival time and polymorphisms in any genetic models (Table 2).

### *MTRR 66A > G* polymorphism on protection of GC patients

To better demonstrate the protective effect of *66A > G* polymorphism on GC survival in detail, we performed stratified analyses based on tumour size, tumour site, histological type, depth of invasion, lymph node metastasis, distant metastasis, TNM stage and chemotherapy. No obvious association was detected with tumor size or location in GC patients (Table 3). Surprisingly, we detected significant protection of *GA + GG* genotypes in patients with more aggressively diffused tumors than in patients with *AA* genotype (MST 70 months vs. 37 months, HR = 0.744, 95% CI = 0.58–0.954, *p* = 0.02). Similar results were obtained in subgroups with advanced tumors. Although there was no detectable protection in T1, N0, M0, TNM stage I/II subgroups, *GA + GG* genotypes significantly prolonged the life of GC patients in subgroups of T2/T2/T3 level depth of invasion (HR = 0.724, 95% CI = 0.580–0.900, *p* = 0.004), N1/N2/N3 level lymph node metastasis (HR = 0.772, 95% CI = 0.607–0.981, *p* = 0.035), no distance metastasis (HR = 0.785, 95% CI = 0.643–0.958, *p* = 0.017) and II/III level TNM stages (HR = 0.771, 95% CI = 0.599–0.994, *p* = 0.045). We also noticed that after treating with 5-Fluorouracil, patients with *GA + GG* genotypes showed longer survival time than those with *AA* genotype (HR = 0.668, 95% CI = 0.470–0.949, *p* = 0.024).

**Table 3:** Stratification analysis of the *MTRR 66A > G* polymorphism and gastric cancer patient's survival

Variables	Genotypes (Dominant model)		MST(AA/GA+GG)	HR(95% CI)^a^	PHeterogeneity
	AA	GA/GG			
**Total(**N **= 885)**	489/242	396/165	51/97	0.793(0.651–0.967)	0.022
**Tumor size**					
≤5 cm	303/139	249/93	70/79^b^	0.773(0.595–1.006)	0.055
>5 cm	186/103	147/72	43/63	0.798(0.589–1.081)	0.145
**Tumor site**					
Non-cardia	327/165	257/109	50/73^b^	0.813(0.639–1.036)	0.094
Cardia	162/77	139/56	52/97	0.751(0.532–1.061)	0.105
**Histological type**					
Intestinal type	205/80	174/64	75^b^/80^b^	0.903(0.650–1.254)	0.542
Diffuse type	284/162	222/101	37/70	0.744(0.580–0.954)	0.02
**Depth of invasion**					
T1	92/28	81/27	85^b^/84^b^	1.114(0.657–1.891)	0.688
T2/T3/T4	386/208	310/134	43/78	0.724(0.582–0.900)	0.004
**Lymph node metastasis**					
N0	190/73	159/50	77^b^/85^b^	0.788(0.550–1.130)	0.195
N1/N2/N3	291/165	234/112	37/65	0.772(0.607–0.981)	0.035
**Distant metastasis**					
M0	481/238	390/162	51/97	0.785(0.643–0.958)	0.017
M1	8/4	5/2	40/27^b^	1.028(0.183–5.759)	0.975
**TNM stage**					
I/II	234/93	187/59	76b/85^b^	0.793(0.551–1.058)	0.105
III/IV	252147	205/103	36/62	0.771(0.599–0.994)	0.045
**Chemotherapy**					
NO 5-FU	349/170	252/110	65/64	0.870(0.684–1.106)	0.254
5-FU	140/72	144/55	51/78^b^	0.668(0.470–0.949)	0.024

a Adjusted for age and sex.

b Heterogeneity test for differences between groups.

c Information was not available for two patients.

### Effects of polymorphism interactions on patient survival

**Figure 2:**
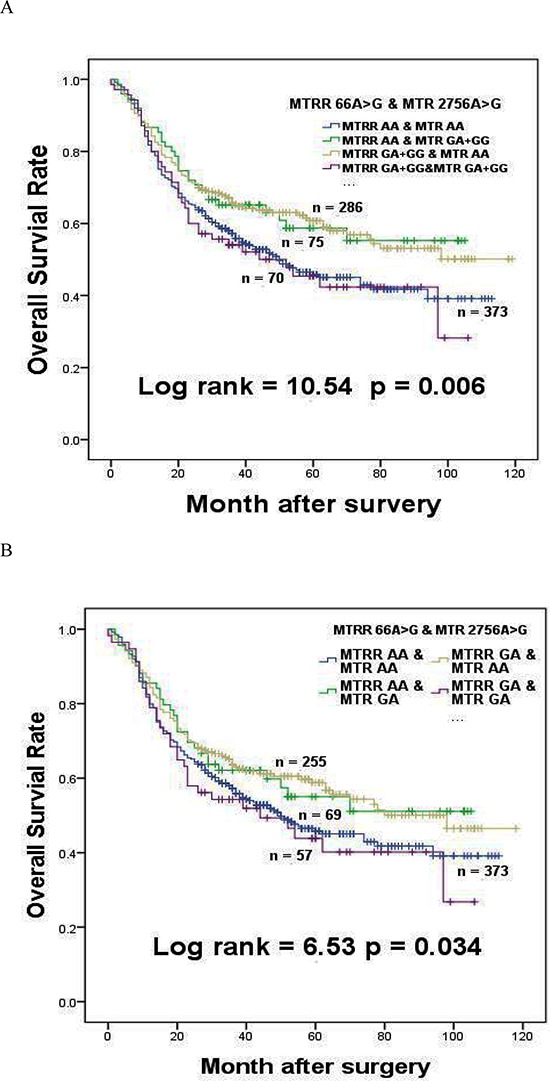
The effects of *MTRR 66A > G* interaction with *MTR 2756A > G* on survival of gastric cancer patients **(A)** The effect of *MTRR* 66A > G interaction with *MTR 2756A > G* in dominant model on the survival of gastric cancer patients. **(B)** The effect of *MTRR 66A > G* interaction with *MTR* 2756A > G in heterozygote model on the survival of gastric cancer patients.

Considering the intricate interactions among the genes described above, we tested polymorphism interactions on the survival of GC patients (Supplementary Table S1–S3). In univariate analysis, *MTRR 66 GA + GG* genotypes significantly decreased risk of death, but the improved survival disappeared when GC patients simultaneously had *MTR 2756 GA + GG* genotypes (HR = 1.063, 95% CI = 0.750–1.507, log-rank *p* = 0.730, Table 4). Interestingly, the protective effect of *MTRR 66 GA + GG* genotypes was maintained in patients with *MTR 2756AA* genotypes (HR = 0.720, 95% CI = 0.569–0.910, log-rank *p* = 0.006). The *MTRR 66GA* genotype was not associated with the survival of GC patients in univariate analysis, but patients with simultaneous *MTRR 66GA* and *MTR 2756AA* genotypes exhibited significant risk reduction of death (HR = 0.773, 95% CI = 0.609–0.981, log-rank *p* = 0.034). However, no significant protective effects were found in patients with *MTRR 66GA* and *MTR 2756GA* genotypes (HR = 1.112, 95% CI = 0.764–1.617, log-rank *p* = 0.580). The effects of *MTRR 66A > G* interaction with *MTR 2756A > G* on the survival of GC patients in dominant model and heterozygote model were shown in Figure 2A and Figure 2B.

**Table 4:** The effects of gene-gene interactions on the survival of gastric cancer patients

combined genotypes	patients	Deaths	MST (months)	*P*	HR (95%CI)^a^
***MTHFR 1298A>C and TS5-UTR 2R>3R***					
MTHFR 1298AA+TS5-UTR 2R2R	26	16	30	0.111	1.00
MTHFR 1298AA+TS5-UTR 2R2R+2R3R	565	262	67	0.790	0.636(0.3840–1.054)
MTHFR 1298CC+CA+TS5-UTR 2R2R	14	8	33	0.510	0.752(0.322–1.757)
MTHFR 1298CC+CA+TS5-UTR2R2R+2R3R	225	91	98	0.022	0.536(0.315–0.913)
***MTRR 66A>G and MTR 2756A>G***					
MTRR 66AA+MTR 2756AA	373	187	50	0.016	1.00
MTRR 66AA+MTR 2756GG+GA	75	30	69^b^	0.082	0.710(0.483–1.044)
MTRR 66GG+GA+MTR 2756AA	286	112	75	0.006	0.720(0.569–0.910)
MTRR 66GG+GA+MTR 2756GG+GA	70	38	52	0.730	1.063(0.750–1.507)
***MTRR 66A>G and MTR 2756A>G***					
MTRR 66AA+MTR 2756AA	373	187	50	0.094	1.00
MTRR 66AA+MTR 2756GA	69	30	65^b^	0.223	0.787(0.535–1.157)
MTRR 66GA+MTR 2756AA	255	106	98	0.034	0.773(0.609–0.981)
MTRR 66GA+MTR 2756GA	57	32	44	0.580	1.112(0.764–1.617)

a Adjusted for age and sex.

b Mean survival time was provided when MST could not be calculated.

**Figure 3:**
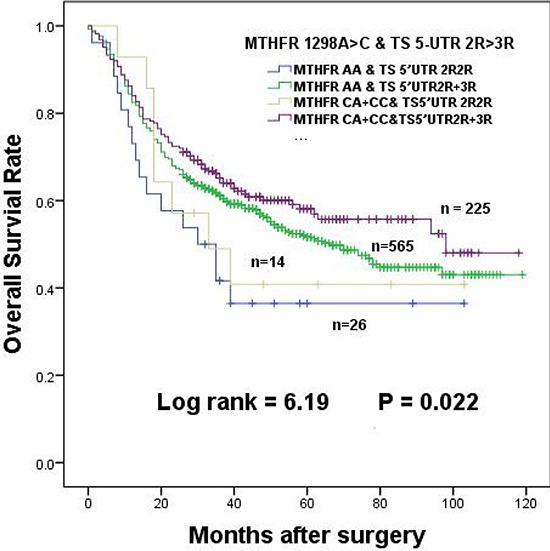
The effects of *MTHFR 1298A > C* interaction with *TS 5-UTR 2R > 3R* in dominant model on the survival of gastric cancer patients

Although the *MTHFR 1298 CA + CC* genotypes were not associated with overall survival in univariate analysis, significant protective effect occurred when the above were combined with *TS* 5′-UTR *2R3R + 3R3R* genotypes (HR = 0.536, 95% CI = 0.315–0.913, log-rank *p* = 0.022, Table 4). Compared to *1298AA* + *TS* 5′-UTR *2R2R* genotypes, MST of patients with *1298 CA + CC* and *TS* 5′-UTR *2R3R + 3R3R* genotypes was extended from 30 months to 98 months (Table 4). The overall survival of GC patients was obviously extended and shown in Figure 3.

## DISCUSSION

In this study, we investigated the effects of polymorphisms and their interactions in OCM pathway on survival of GC patients. The results demonstrated that the polymorphisms of *MTRR 66A > G* and *MTHFR 1298A > C* increased the survival of GC in a Chinese population. The MST of GC patients were strongly affected by *MTHFR 1298A > C*, *TS* 5′-UTR copy numbers and interactions between *MTRR 66A > G* and *MTR 2756A > G*.

DNA dysmethylation is one of the crucial events for cancer development. It is well known that hypermethylation on promoters of tumor suppressor genes causes human cancers. *MTRR* and *MTR* maintained adequate levels of methionine, which functioned as a precursor for the universal methyl group donor S-adenosylmethionine [12]. The mutation of *MTRR 66A > G* led to abnormal DNA methylation and altered nucleotide synthesis and repair [21]. *MTRR 66A > G* polymorphism has been shown to increase risk of esophageal, colorectal and lung cancer [11, 12, 14]. However, some groups reported that the polymorphism had no significant effect on colorectal and breast cancers or cervical cancer [28, 29]. Our present result showed that *MTRR 66A > G* polymorphism displayed a protective effect on GC among Chinese population (Table 2 and Figure 1), which was similar to the results in prostate cancer and colon cancer [30, 31]. Clinical analysis for functional contribution of polymorphism with multiple subgroups on the basis of clinical characteristics was vital to identify the potential prognostic markers in diverse stages of cancer progression. Several other studies have also demonstrated that the clinical characteristics of GC were related to the survival of GC patients [32, 33]. Our findings indicated that patients with *MTRR 66 GA + GG* genotypes had better survival among subgroups that have more pronounced diffuse type, greater depth of invasion (T2/T3/T4), higher-level lymph node metastasis (N1/N2/N3), advanced TNM stages (III/IV), and 5-FU treatment. The results indicated that *MTRR 66G* allele improved the survival of GC patients. It has been proposed that *MTR* activity could not be maintained at a high level because residue Ile22 to Met22 switch of *MTRR* caused by the polymorphism reduced it activity [34]. Therefore, *MTR* could not effectively generate methionine for DNA methylation. We postulated that reduced methylation on promoters of tumor suppressor genes would finally decrease activities of GC cells such as cell growth, invasiveness.

Our knowledge on the association of cancer risk and *MTR 2756A > G* polymorphism is very limited and inconsistent. Marcella et al. showed that *MTR* 2756*GG* genotype associated with decreased risk of colorectal cancer death [35]. A meta-analysis showed that *MTR 2756GG* genotype obviously increased cancer risk in Asian populations, whereas a significantly reduced risk was observed in European populations [36]. Some other studies, consistent with our result among GC patients, demonstrated that no association was identified in colon cancer risk [37, 38]. In this study, we did not observe significant association of *GG* genotype with survival time in GC patients (Table 2). Surprisingly, we found *MTR 2756 GA + GG* polymorphisms (Asp868 to Gly868 switch) neutralized the effects of *MTRR 66 GA + GG* on prolonged survival of GC patients (Table 4, Figure 2). Although there is no crystal structure data on *MTRR-MTR* interactions, based on our data, we could deduce that Ile22 of *MTRR* and Asp868 of *MTR* are crucial for *MTRR-MTR* interactions. Met22 switch of *MTRR* may impair *MTRR-MTR* interaction, while three-dimensional conformation change caused by Gly868 switch of *MTR* may at least partially compensate and restore their interactions.

The polymorphisms of *MTHFR 1298A > C* and *677C > T* were reported to reduce MTHFR enzymatic activity, leading to an increase in the amount of 5,10-MTHF [19]. 5,10-MTHF, a substrate for both TS and MTHFR, interacted with TS and FU to form a tertiary complex, which might strengthen the function of 5-FU. 5-FU, the most common chemotherapy drug, was used to treat many cancers including GC. TS was a primary target for 5-FU. It was reported that polymorphism of both *MTHFR 1298A > C* and *677C > T* manifestly protected the survival of colorectal cancer patients [39]. Recent studies have demonstrated that *677T* allele significantly increased the protective effect on colorectal cancer and hepatocellular cancer patients [35, 40, 41]. However, even as the polymorphisms cause a glutamine-to-alanine switch in protein sequence, we found that only *MTHFR 1298CC* genotype showed a comparatively favorable outcome in this study, indication of the protection of GC patient survival in a recessive way. And although polymorphisms of *TS* have been proposed to influence 5-FU sensitivity, the accumulated data were controversial. One study showed that patients with *TS* 5′-UTR *3R3R* genotype had poor prognosis in neoadjuvant treated advanced GC [42], while several other studies did not reveal any obviously prognostic differences among patients [43–45]. In term of *TS* 3′-UTR polymorphisms, results from different groups were also not consistent. Some showed an increase of survival for colorectal cancer patients treated with 5-FU based adjuvant chemotherapy [46, 47], whereas others reported no obvious difference [43, 48]. In this study, no obvious relationship was found between the polymorphisms of *TS* 3′-UTR and 5-UTR genotypes with patient survival (Table 2). Interestingly, we observed *TS* 5′UTR *2R3R + 3R3R* genotypes could interact with *MTHFR 1298 CA + CC* polymorphisms. They jointly reduced the risk of death of GC patients although each did not show detectable effect independently (Table 4). Patients carrying *MTHFR 1298AA* and *TS* 5-UTR *2R2R* genotypes showed a relatively poor prognosis as compared to those with *MTHFR 1298 CA + CC* and *TS* 5-UTR *2R3R + 3R3R* genotypes. Because *3R* exhibits better translational efficiency than *2R*, more 5,10-MTHF, TS and 5-FU complexes could be formed; it also displays better protective effect on GC progression to a certain extent [49].

In summary, our findings showed that *MTHFR 1298A > C* and *MTRR 66A > G* polymorphisms were related to the survival of GC patients, which indicated that these polymorphisms may be useful biomarkers for more accurate assessments of GC prognosis in the Chinese population. Further investigations on gene-gene interactions that may enhance or neutralize the effects of each individual polymorphism are needed.

## PATIENTS AND METHODS

### Patient samples

All 919 GC patients were recruited at the Yixing People's Hospital (Yixing, Jiangsu Province, China) between January 1999 and December 2006. They were histopathologically diagnosed and underwent surgery. No patient had received radiotherapy or chemotherapy before surgery. The patients’ clinical characteristics were summarized in Table 1, with the maximum follow-up time of 119.0 months and the median follow-up time of 68.5 months. We classified the histopathology of GC into intestinal or diffuse types according to Lauren's Standard [50]. The TNM stages were evaluated according to the TNM classification of the American Joint Committee on Cancer (AJCC cancer staging manual, seventh edition). The retrospective cohort study was approved by the Institutional Review Board of Nanjing Medical University (Nanjing, China). All patients signed an informed consent on using clinical specimen for medical research in this study.

### Determination of polymorphism incidences

Genomic DNA was extracted from paraffin sections postoperative tissues by proteinase K digestion, isopropanol extraction, and ethanol precipitation [32]. Polymorphisms were measured by SNaPshot technology with an ABI fluorescence-based assay allelic discrimination method (Applied Biosystems, Forster City, CA) or automated sequencing as previously described [51]. Primers were custom made and their sequences were listed in Supplementary Table S4. An ABI3130 genetic analyzer was used to analyze the polymorphisms, and Genemapper4.0 software was used to determine genotypes (Applied Biosystems). Two people independently performed genotyping assays in a blind fashion. 10% random samples were selected to validate genotypes, and the results were 100% concordant.

### Statistical analysis

Mean survival time was used in this study if the median survival time (MST) could not be calculated. The overall survival time of each patient was calculated from the date of surgery until death or the last follow-up (March 31, 2009). The correlations of each genotype and combinations of genotypes with clinicopathologic parameters were compared. Student *t*-test and the Pearson were employed in this study based on the types of variables. Survival analyses were carried out by Kaplan–Meier plots and log-rank tests in SPSS version 20.0 (SPSS Inc, Chicago, IL, USA). We used univariate or multivariate Cox regression analysis to estimate 95% confidence intervals (CIs), and crude/adjusted hazard ratios (HRs). All of the tests were two-sided and *p* < 0.05 was considered statistically significant.

## SUPPLEMENTARY FIGURES


