# Supplementary material for: Polymorphism in one-carbon metabolism pathway affects survival of gastric cancer patients: Large and comprehensive study
Source: Oncotarget. 2015 Mar 25;6(11):9564–76. doi: 10.18632/oncotarget.3259 (PMC4496239; doi:10.18632/oncotarget.3259)
Supplement: Supplementary file 1 [file oncotarget-06-9564-s001.pdf]

SUPPLEMENTARY TABLES

Supplementary Table S1: The effects of gene-gene interactions in homozygous model on the survival of gastric cancer patients

combined genotypes	patient	death	MST	*P*	HR (95%CI)[a]
MTRR 66A>G and MTHFR 1298A>C					
MTRR 66AA + MTHFR 1298AA	338	171	41	0.135	1
MTRR 66AA + MTHFR 1298CC	16	5	68	0.187	0.550(0.226–1.3380
MTRR 66GG + MTHFR 1298AA	37	11	93	0.044	0.535(0.291–0.985)
MTRR 66GG + MTHFR 1298CC	1	0	0	0.952	0
MTRR 66A>G and MTHFR 677C>T					
MTRR 66AA + MTHFR 677CC	171	87	49	0.169	1
MTRR 66AA + MTHFR 677TT	73	34	54	0.513	0.876(0.589–1.302)
MTRR 66GG + MTHFR 677CC	13	3	95[b]	0.043	0.305(0.096–0.965)
MTRR 66GG + MTHFR 677TT	8	5	31[b]	0.472	1.393(0.565–3.435)
MTRR 66A>G and MTR 2756A>G					
MTRR 66AA + MTR 2756AA	373	187	76	0.25	1
MTRR 66AA + MTR 2756GG	6	0	0	0.954	0
MTRR 66GG + MTR 2756AA	31	6	89	0.007	0.324(0.143–0.730)
MTRR 66GG + MTR 2756GG		0	0	0	0
***MTRR 66A>G and TS5*–UTR 2R>3R**					
MTRR 66AA + TS5–UTR 2R2R	21	11	39	0.133	1
MTRR 66AA + TS5-UTR 3R3R	276	134	51	0.999	0.999(0.540–1.850)
MTRR 66GG + TS5–UTR 2R2R	3	2	26	0.65	1.417(0.314–6.404)
MTRR 66GG + TS5-UTR 3R3R	33	8	91	0.072	0.433(0.174–1.077)
***MTRR 66A>G and TS3*-UTR I>D**					
MTRR 66AA + TS3-UTRII	32	16	46	0.241	1
MTRR 66AA + TS3-UTR DD	223	108	52	0.838	0.947(0.560–1.601)
MTRR 66GG + TS3-UTR II	5	3	26	0.792	1.180(0.344–4.055)
MTRR 66GG + TS3-UTR DD	23	5	92[b]	0.062	0.384(0.140–1.048)
MTHFR 1298A>C and MTHFR 677C>T					
MTHFR 1298AA + MTHFR677CC	165	88	48	0.1	1
MTHFR 1298AA + MTHFR677TT	137	61	76	0.154	0.789(0.569–1.093)
MTHFR 1298CC + MTHFR677CC	26	7	92	0.023	0.410(0.190–0.886)
MTHFR 1298CC + MTHFR677TT	1	0	0	0.951	0
MTHFR 1298A>C and MTR 2756A>G					
MTHFR 1298AA + MTR2756AA	488	227	75	0.435	1
MTHFR 1298AA + MTR2756GG	4	0	0	0.943	0
MTHFR 1298CC + MTR2756AA	21	6	63	0.099	0.505(0.224–1.137)

(*Continued*)

combined genotypes	patient	death	MST	*P*	HR (95%CI)[a]
MTHFR 1298CC + MTR2756GG	1	0	0	0.97	0
MTHFR 1298A>C and TS5-UTR 2R>3R					
*MTHFR 1298AA + TS*5-UTR 2R2R	26	16	30	0.132	1
*MTHFR 1298AA+ TS*5-UTR 3R3R	369	170	65	0.085	0.637(0.381–1.064)
*MTHFR 1298CC + TS*5-UTR 2R2R	3	1	75[b]	0.274	0.323(0.043–2.444)
*MTHFR 1298CC+ TS*5-UTR 3R3R	18	5	74[b]	0.035	0.340(0.124–0.928)
***MTHFR 1298A>C and TS* 3-UTR I>D**					
*MTHFR 1298AA+ TS*3-UTR II	43	23	39	0.303	1
*MTHFR 1298AA + TS*3-UTR DD	288	134	65	0.435	0.838(0.538–1.305)
*MTHFR 1298CC + TS*3-UTR II	3	1	75	0.53	0.526(0.071–3.900)
*MTHFR 1298CC+ TS*3-UTR DD	14	3	73	0.066	0.324(0.097–1.079)
MTHFR 677C>T and MTR 2756A>G					
MTHFR 677CC+ MTR2756AA	238	112	59	0.679	1
MTHFR 677CC+ MTR2756GG	3	0	0	0.951	0
MTHFR 677TT+ MTR2756AA	115	45	65	0.219	0.805(0.569–1.138)
MTHFR 677TT+ MTR2756GG	1	0	0	0.976	0
***MTHFR 677C>T and TS*5-UTR 2R>3R**					
*MTHFR 677CC + TS*5-UTR 2R2R	16	9	39	0.709	1
*MTHFR 677CC+ TS*5-UTR 3R3R	172	76	67	0.573	0.819(0.409–1.639)
*MTHFR 677TT+ TS*5-UTR 2R2R	7	3	58[b]	0.708	0.779(0.211–2.882)
*MTHFR 677TT + TS*5-UTR 3R3R	80	31	78	0.298	0.673(0.319–1.418)
***MTHFR 677C>T and TS*3-UTR I>D**					
*MTHFR 677CC+ TS*3-UTRII	24	12	46	0.758	1
*MTHFR 677CC + TS*3-UTR DD	141	65	77	0.577	0.839(0.453–1.554)
*MTHFR 677TT + TS*3-UTR II	6	2	69[b]	0.319	0.467(0.104–2.090)
*MTHFR 677TT + TS*3-UTR DD	61	27	63	0.478	0.782(0.386–1.544)
***MTR 2756A>G and TS*5-UTR 2R>3R**					
*MTR 2756AA + TS*5-UTR 2R2R	24	12	47	0.825	1
*MTR 2756AA+ TS*5-UTR 3R3R	393	169	59	0.537	0.831(0.463–1.493)
*MTR 2756 GG + TS*5-UTR 2R2R	0	0	0	0.947	0
*MTR 2756 GG + TS*5-UTR 3R3R	4	0	0	0	0
***MTR 2756A> G and TS*3-UTR I>D**					
*MTR 2756AA + TS*3-UTRII	43	20	76	0.936	1
*MTR 2756AA + TS*3-UTR DD	304	136	59[b]	0.72	0.918(0.574–1.468)
*MTR 2756 GG + TS*3-UTR II	4	0	0	0.949	0
*MTR 2756 GG + TS*3-UTR DD	0	0	0	0	0

(*Continued*)

combined genotypes	patient	death	MST	*P*	HR (95%CI)[a]
***TS3-UTR I>D and TS* 5-UTR 2R>3R**					
*TS*3-UTR II+ *TS*5-UTR 2R2R	14	9	20	0.777	1
*TS*3-UTR II+ *TS*5-UTR 3R3R	10	5	46[b]	0.482	0.674(0.225–2.021)
*TS*3-UTR DD+ *TS*5-UTR 2R2R	0	0	0	0	0
*TS*3-UTR DD+ *TS*5-UTR 3R3R	6	3	14	0.902	0.921(0.249–3.409)

[a]Adjusted for age and sex.
[b]Mean survival time was provided when MST could not be calculated.

Supplementary Table S2: The effects of gene-gene interactions in heterozygous model on the survival of gastric cancer patients

combined genotypes	patient	death	MST	*P*	HR (95%CI)[a]
MTRR 66A>G and MTHFR 1298A>C					
MTRR 66AA + MTHFR 1298AA	338	171	50	0.436	1
MTRR 66AA + MTHFR 1298CA	127	61	53	0.723	0.949(0.708–1.271)
MTRR 66GA + MTHFR 1298AA	240	106	78	1.41	0.833(0.654–1.062)
MTRR 66GA + MTHFR 1298CA	91	40	98	0.281	0.827(0.586–1.168)
MTRR 66A>G and MTHFR 677C>T					
MTRR 66AA + MTHFR 677CC	171	87	49	0.457	1
MTRR 66AA + MTHFR 677TC	236	118	50	0.742	0.955(0.724–1.259)
MTRR 66GA + MTHFR 677CC	117	53	77	0.328	0.843(0.599–1.186)
MTRR 66GA + MTHFR 677TC	161	69	97	0.156	0.795(0.580–1.091)
MTRR 66A>G and MTR 2756A>G					
MTRR 66AA + MTR 2756AA	373	187	50	0.094	1
MTRR 66AA + MTR 2756GA	69	30	66[b]	0.223	0.787(0.535–1.157)
MTRR 66GA + MTR 2756AA	255	106	98	0.034	0.773(0.609–0.981)
MTRR 66GA + MTR 2756GA	57	32	44	0.58	1.112(0.764–1.617)
*MTRR 66A>G and TS*5-UTR *2R>3R*					
*MTRR 66AA + TS*5-UTR *2R2R*	21	11	39	0.4	1
*MTRR 66AA + TS*5-UTR *2R3R*	149	75	51	0.807	1.082(0.574–2.039)
*MTRR 66GA + TS*5-UTR *2R2R*	17	10	30	0.401	1.444(0.612–3.404)
*MTRR 66GA + TS*5-UTR *2R3R*	104	45	88	0.631	0.851(0.440–1.645)
*MTRR 66A>G and TS*3-UTR I>D					
*MTRR 66AA + TS*3-UTRII	32	16	46	0.581	1
*MTRR 66AA + TS*3-UTR DI	218	111	49	0.974	1.009(0.597–1.704)
*MTRR 66GA + TS*3-UTR II	24	11	57[b]	0.691	0.856(0.397–1.845)
*MTRR 66GA + TS*3-UTR DI	156	67	88	0.475	0.820(0.475–1.414)

(*Continued*)

combined genotypes	patient	death	MST	*P*	HR (95%CI)[a]
MTHFR 1298A>C and MTHFR 677C>T					
MTHFR 1298AA + MTHFR677CC	165	88	48	0.413	1
MTHFR 1298AA + MTHFR677TC	323	146	78	0.117	0.809(0.621–1.054)
MTHFR 1298CA + MTHFR677CC	120	54	63	0.187	0.796(0.567–1.117)
MTHFR 1298CA + MTHFR677TC	110	52	94	0.367	0.854(0.606–1.203)
MTHFR 1298A>C and MTR 2756A>G					
MTHFR 1298AA + MTR2756AA	488	227	65	0.987	1
MTHFR 1298AA + MTR2756GA	97	49	54	0.758	1.050(0.771–1.430)
MTHFR 1298CA + MTR2756AA	174	81	63	0.96	0.994(0.771–1.280)
MTHFR 1298CA + MTR2756GA	39	18	64[b]	0.881	0.964(0.597–1.558)
*MTHFR 1298A>C and TS*5-UTR 2R>3R					
*MTHFR 1298AA + TS*5-UTR 2R2R	26	16	30	0.351	1
*MTHFR 1298AA + TS*5-UTR 2R3R	196	92	70	0.097	0.638(0.375–1.085)
*MTHFR 1298CA + TS*5-UTR 2R2R	11	7	33	0.819	0.901(0.371–2.192)
*MTHFR 1298CA + TS*5-UTR 2R3R	71	34	62	0.262	0.711(0.392–1.289)
*MTHFR 1298A>C and TS*3-UTR I>D					
*MTHFR 1298AA + TS*3-UTRII	43	23	39	0.925	1
*MTHFR 1298AA + TS*3-UTR DI	294	139	67	0.572	0.881(0.566–1.369)
*MTHFR 1298CA + TS*3-UTR II	15	6	55	0.554	0.762(0.310–1.873)
*MTHFR 1298CA + TS*3-UTR DI	102	49	66	0.655	0.893(0.544–1.466)
MTHFR 677C>T and MTR 2756A>G					
MTHFR 677CC+ MTR2756AA	238	112	62	0.734	1
MTHFR 677CC+ MTR2756GA	46	22	60[b]	0.795	1.063(0.673–1.679)
MTHFR 677TC+ MTR2756AA	321	151	74	0.736	1.043(0.817–1.332)
MTHFR 677TC+ MTR2756GA	74	32	97	0.391	0.842(0.568–1.247)
*MTHFR 677C>T and TS*5-UTR 2R>3R					
*MTHFR 677CC + TS*5-UTR 2R2R	16	9	39	0.389	1
*MTHFR 677CC+ TS*5-UTR 2R3R	97	48	52	0.776	0.902(0.442–1.838)
*MTHFR 677TC+ TS*5-UTR 2R2R	17	11	33	0.392	1.495(0.618–3.615)
*MTHFR 677TC + TS*5-UTR 2R3R	129	59	74	0.652	0.851(0.422–1.716)
*MTHFR 677C>T and TS*3-UTR I>D					
*MTHFR 677CC+ TS*3-UTRII	24	12	46	0.791	1
*MTHFR 677CC + TS*3-UTR DI	135	67	56	0.945	0.978(0.529–1.809)
*MTHFR 677TC + TS*3-UTR II	31	16	38	0.909	1.045(0.494–2.208)
*MTHFR 677TC + TS*3-UTR DI	195	90	74	0.616	0.857(0.469–1.565)

(*Continued*)

combined genotypes	patient	death	MST	*P*	HR (95%CI)[a]
*MTR 2756A>G and TS*5-UTR 2R>3R					
MTR 2756AA + *TS*5-UTR 2R2R	24	12	39	0.13	1
MTR 2756AA + *TS*5-UTR 2R3R	216	104	56	0.948	0.980(0.539–1.782)
MTR 2756GA + *TS*5-UTR 2R2R	10	8	18	0.097	2.135(0.872–5.232)
MTR 2756GA + *TS*5-UTR 2R3R	35	15	70[b]	0.499	0.769(0.360–1.645)
*MTR 2756A>G and TS*3-UTR I>D					
MTR 2756AA + *TS*3-UTRII	43	20	55[b]	0.938	1
MTR 2756AA + *TS*3-UTR DI	317	149	63	0.887	1.035(0.649–1.650)
MTR 2756GA + *TS*3-UTR II	16	9	26	0.642	1.205(0.549–2.647)
MTR 2756GA + *TS*3-UTR DI	54	25	70	0.86	0.949(0.527–1.708)
*TS*3-UTR I>D and *TS*5-UTR 2R>3R					
*TS*3-UTR II+ *TS*5-UTR 2R2R	14	9	20	0.631	1
*TS*3-UTR II+ *TS*5-UTR 2R3R	35	15	62[b]	0.279	0.633(0.277–1.449)
*TS*3-UTR DD+ *TS*5-UTR 2R2R	21	12	35	0.795	0.891(0.375–2.119)
*TS*3-UTR DD+ *TS*5-UTR 2R3R	180	86	70	0.33	0.711(0.357–1.414)

[a]Adjusted for age and sex.
[b]Mean survival time was provided when MST could not be calculated.

Supplementary Table S3: The effects of gene-gene interactions in dominant model on the survival of gastric cancer patients

combined genotypes	patient	death	MST	*P*	HR (95%CI)[a]
MTRR 66A>G and MTHFR 1298A>C					
MTRR 66AA + MTHFR 1298AA	338	171	50	0.106	1
MTRR66AA+MTHFR1298CC+CA	143	66	94	0.458	0.898(0.676–1.193)
MTRR 66GG+GA + MTHFR 1298AA	277	117	80	0.053	0.793(0.627–1.003)
MTRR66GG+GA+MTHFR1298CC+CA	113	45	76	0.042	0.712(0.512–0.988)
MTRR 66A>G and MTHFR 677C>T					
MTRR 66AA + MTHFR 677CC	171	87	49	0.089	1
MTRR 66AA + MTHFR 677TT+TC	309	152	52	0.626	0.937(0.720–1.219)
MTRR 66GG+GA + MTHFR 677CC	130	56	98	0.125	0.769(0.549–1.076)
MTRR 66GG+GA + MTHFR 677TT+TC	253	101	75[b]	0.031	0.729(0.547–0.971)
MTRR 66A>G and MTR 2756A>G					
MTRR 66AA + MTR 2756AA	373	187	50	0.016	1
MTRR 66AA + MTR 2756GG+GA	75	30	69b	0.082	0.710(0.483–1.044)
MTRR 66GG+GA + MTR 2756AA	286	112	76[b]	0.006	0.720(0.569–0.910)
MTRR 66GG+GA + MTR 2756GG+GA	70	38	57[b]	0.73	1.063(0.750–1.507)
***MTRR 66A>G and TS* 5-UTR 2R>3R**					
*MTRR 66AA + TS*5-UTR 2R2R	21	11	39	0.011	1

(*Continued*)

combined genotypes	patient	death	MST	*P*	HR (95%CI)[a]
MTRR 66AA + *TS*5-UTR 2R2R+2R3R	425	209	51	0.892	1.043(0.568–1.913)
MTRR 66GG+GA + *TS*5-UTR 2R2R	20	12	26	0.347	1.482(0.653–3.361)
*MTRR66GG+GA+TS*5-UTR2R2R+2R3R	342	134	97	0.37	0.755(0.408–1.396)
***MTRR 66A>G and TS* 3-UTR I>D**					
MTRR 66AA + *TS*3-UTR II	32	16	46	0.103	1
MTRR66AA + *TS*3-UTR II+DI	441	219	51	0.93	0.978(0.588–1.624)
MTRR 66GG+GA + *TS*3-UTR II	29	14	56[b]	0.821	0.920(0.449–1.886)
MTRR 66GG+GA + *TS*3-UTR II+DI	351	144	97	0.281	0.753(0.449–1.262)
MTHFR 1298A>C and MTHFR 677C>T					
MTHFR 1298AA + *MTHFR 677CC*	165	88	48	0.215	1
MTHFR 1298AA + *MTHFR 677TT+TC*	460	207	74	0.086	0.804(0.626–1.032)
MTHFR 1298CC+CA + *MTHFR 677CC*	146	61	98	0.05	0.721(0.520–1.000)
MTHFR1298CC+CA+MTHFR677TT+TC	113	52	94	0.262	0.822(0.583–1.158)
MTHFR 1298A>C and MTR 2756A>G					
MTHFR 1298AA + *MTR 2756AA*	488	227	65	0.836	1
MTHFR 1298AA + *MTR2756GG+GA*	101	49	62	0.968	0.994(0.730–1.353)
MTHFR 1298CC+CA + *MTR 2756AA*	195	87	89	0.577	0.932(0.728–1.193)
MTHFR1298CC+CA+MTR2756GG+GA	47	19	68[b]	0.426	0.827(0.518–1.321)
***MTHFR 1298A>C and TS* 5-UTR 2R>3R**					
MTHFR 1298AA + *TS*5-UTR 2R2R	26	16	30	0.111	1
*MTHFR1298AA+TS*5-UTR 2R2R+2R3R	565	262	67	0.079	0.636(0.384–1.054)
MTHFR 1298CC+CA+ TS 5-UTR 2R2R	14	8	33	0.51	0.752(0.322–1.757)
*MTHFR1298CC+CA+TS*5-UTR2R2R+2R3R	225	91	98	0.022	0.536(0.315–0.913)
***MTHFR 1298A>C and TS* 3-UTR I>D**					
MTHFR 1298AA + *TS*3-UTR II	43	23	39	0.674	1
*MTHFR 1298AA+ TS*3-UTR DD+DI	582	273	65	0.479	0.858(0.560–1.313)
MTHFR 1298CC+CA + *TS*3-UTR II	18	7	68	0.441	0.717(0.308–1.672)
MTHFR 1298CC+CA + *TS*3-UTR DD+DI	234	103	71	0.284	0.781(0.497–1.227)
MTHFR 677C>T and MTR 2756A>G					
MTHFR 677CC+ MTR2756AA	238	112	62	0.996	1
MTHFR 677CC+ MTR2756GG+GA	49	22	63[b]	0.906	0.973(0.616–1.537)
MTHFR 677TT+TC + *MTR2756AA*	436	196	74	0.837	.976(0.774–1.231)
MTHFR 677TT+TC + *MTR2756GG+GA*	99	47	70	0.833	0.964(0.686–1.355)
***MTHFR 677C>T and TS* 5-UTR 2R>3R**					
MTHFR 677CC + *TS*5-UTR 2R2R	16	9	39[b]	0.336	1
*MTHFR 677CC+ TS*5-UTR 2R2R+2R3R	269	124	63	0.628	0.846(0.430–1.665)

(*Continued*)

combined genotypes	patient	death	MST	*P*	HR (95%CI)[a]
*MTHFR 677TT+TC+ TS*5-UTR 2R2R	24	14	33	0.567	1.277(0.552–2.955)
*MTHFR677TT+TC+TS*5-UTR2R2R+2R3R	511	225	78	0.499	0.794(0.408–1.548)
***MTHFR 677C>T and TS* 3-UTR I>D**					
*MTHFR 677CC+ TS*3-UTRII	24	12	46	0.895	1
*MTHFR 677CC + TS*3-UTR DD+DI	276	132	62	0.754	0.910(0.504–1.643)
*MTHFR 677TT+TC + TS*3-UTR II	37	18	59	0.842	0.928(0.447–1.927)
*MTHFR 677TT+TC + TS*3-UTR DD+DI	525	237	74	0.594	0.854(0.478–1.526)
***MTR 2756A>G and TS* 5-UTR 2R>3R**					
*MTR 2756AA + TS*5-UTR 2R2R	24	12	39	0.077	1
*MTR 2756AA + TS*5-UTR 2R2R+2R3R	609	273	74	0.671	0.882(0.495–1.573)
*MTR 2756GG+GA + TS*5-UTR 2R2R	10	8	18	0.111	2.073(0.846–5.078)
*MTR2756GG+GA+TS*5-UTR2R2R+2R3R	127	54	97	0.43	0.777(0.416–1.454)
**MTR 2756A>G and TS 3-UTR I>D**					
MTR 2756 AA + TS3-UTRII	43	20	55[b]	0.88	1
MTR 2756 AA + TS3-UTR DD+DI	621	285	67	0.909	0.974(0.619–1.533)
MTR 2756GG+GA + TS3-UTR II	16	9	26	0.684	1.178(0.536–2.587)
MTR 2756GG+GA + TS3-UTR DD+DI	130	58	97	0.69	0.902(0.542–1.499)
***TS* 3-UTR I>D and *TS* 5-UTR 2R>3R**					
*TS*3-UTR II+ *TS*5-UTR 2R2R	14	9	20	0.403	1
*TS*3-UTR II+ *TS*5-UTR 2R3R+3R3R	45	20	62[b]	0.288	0.653(0.297–1.435)
*TS*3-UTR DD+DI + *TS*5-UTR 2R2R	27	15	35	0.756	0.877(0.384–2.005)
*TS*3-UTRDD+DI+ *TS*5-UTR 2R3R+3R3R	747	336	74	0.196	0.646(0.333–1.252)

[a]Adjusted for age and sex.
[b]Mean survival time was provided when MST could not be calculated.

Supplementary Table S4: Primers for the genotyping assays of *MTRR, MTHFR, MTR* and *TS*

Genotypes	Forward primer	Reverse primer	Extension primer
MTRR rs1801394 66*A>G*	5′-ACACAGCAGGGACA GGCAAAG -3′	5′-GCAGAAAATCCA TGTACCACAGC -3′	5′-TTTTTTTTTTTTTTAAG GCCATCGCAGAAGAA AT -3′
MTHFR rs1801131 *129 8 A >C*	5′-AAGGAGGAGCTGC TGAAGATGTG -3′	5′-TGGTTCTCCCGAGA GGTAAAGAACA -3′	5′-TTTTTTTTTTTTTTTTTTT TTTTTTTTTTTTGGTAAAG AACRAAGACTTCAAAGA CACKT -3′
MTHFR rs1801133 677 C> T	5′-TGAGGCTGACCTG AAGCACTTG -3′	5′-CAAAGAAAAGCTG CGTGATGATGA -3′	5′-TTTTTTTTGCTGCGTGA TGATGAAATCG -3′
MTR rs1805087 *2756 A >G*	5′-AAGGATGAATACTT TGAGGAAATCATGG -3′	5′-CTGTTTCTACCACTT ACCTTGAGA GACTCAT -3′	5′-TTTTTTTTTTTTTTTTTT TCATGGAAGAATATGAA GATATTAGACAGG -3′
TYMS 5-UTR *2R > 3R*	5′-CGGAAGGGGTCCTGC CACC -3′	5′-GAGCCGGCCACAG GCATGG -3′	
TYMS 3-UTR *6bp ins>del*	5′-GGAGCTGAGTAACAC CATCGATCA -3′	5′-GCGTGGACGAATGC AGAACA -3′	
